# Sirenomelia With Complete Caudal Regression in a Preterm Infant Born to a Mother With Poorly Controlled Type 1 Diabetes Mellitus: A Case Report on Clinical Presentation and Perinatal Management Challenges

**DOI:** 10.7759/cureus.99620

**Published:** 2025-12-19

**Authors:** Ibrahim Salih, Nasser Al Shafouri, Mahmoud Khalid, Ahmed Al Muqarshi

**Affiliations:** 1 Pediatrics, Ibri Hospital, Ibri, OMN; 2 Pediatrics and Neonatology, Ibri Hospital, Ibri, OMN

**Keywords:** caudal regression sequence, maternal diabetes mellitus, mermaid syndrome, neonatal palliative care, sirenomelia

## Abstract

Sirenomelia is a rare, lethal congenital anomaly characterized by caudal regression and lower limb fusion. The etiopathogenesis is multifactorial, with maternal glycemic dysregulation established as a significant risk factor, contributing to the development of this severe malformation syndrome.

We report an infant born at 33 weeks' gestation with a birth weight of 1,900 g to a 36-year-old multiparous woman with poorly controlled type 1 diabetes mellitus (HbA1c, 9.8%). Prenatal ultrasound evaluation during the second trimester identified significant fetal abnormalities consistent with a lethal congenital malformation syndrome. The neonate presented with complete lower limb fusion, bilateral renal agenesis, imperforate anus, ambiguous genitalia, and dysmorphic facial features. Despite supportive palliative care, the infant died at 24 hours of life due to complications of bilateral renal agenesis and associated malformations.

This case emphasizes the critical importance of optimal preconception glycemic control in diabetic women and highlights the challenges in prenatal counseling and neonatal management of sirenomelia. The case contributes to the growing evidence linking poor maternal glycemic control with severe caudal regression anomalies and underscores the need for enhanced periconceptional counseling.

## Introduction

Sirenomelia, also known as mermaid syndrome or caudal regression sequence, is a specific and distinct form of caudal regression characterized by complete lower limb fusion. While "caudal regression syndrome" is a broader umbrella term encompassing various degrees of sacral agenesis and lower limb abnormalities, sirenomelia represents the most severe end of this spectrum, defined by fusion of the lower extremities into a single structure. The condition occurs in approximately 0.8-1.0 per 100,000 live births, with a male predominance (male:female ratio, 2.7:1) [1,2].

The phenotypic spectrum of sirenomelia ranges from mild lower limb fusion with preserved renal function to severe forms with complete caudal regression, bilateral renal agenesis, and multiple visceral malformations [3]. The most severe presentations, characterized by complete caudal regression and bilateral renal agenesis, are invariably fatal due to oligohydramnios-induced pulmonary hypoplasia and anuria. Prenatal diagnosis of sirenomelia is typically achieved through second- and third-trimester ultrasound; however, severe oligohydramnios, associated with bilateral renal agenesis, can limit sonographic visualization and delay diagnosis until a later gestational age. This diagnostic constraint has important implications for the timing of prenatal counseling and family decision-making.

Maternal diabetes mellitus has emerged as the most consistently documented risk factor, reported in 15%-22% of affected pregnancies, with diabetic mothers having a 200-fold increased risk compared to the general population [4]. The teratogenic effect appears related to poor glycemic control during the critical period of caudal mesoderm development (four to seven weeks of gestation) [5].

## Case presentation

A 36-year-old woman, G6P4A1, with poorly controlled type 1 diabetes mellitus, presented at 33 weeks of gestation. She had been diagnosed with type 1 diabetes one year prior and demonstrated persistently suboptimal glycemic control throughout the pregnancy and beyond. Her HbA1c at eight weeks’ gestation was 9.8% (equivalent to average glucose >250 mg/dL), indicating severe hyperglycemia during the critical organogenesis period. Serial monitoring showed HbA1c of 8.0% at 12 weeks and 8.7% at 20 weeks, demonstrating ongoing poor control despite medical intervention. There was no family history of congenital anomalies or known teratogen exposure during the pregnancy.

Prenatal ultrasound evaluation during the second trimester identified significant fetal abnormalities consistent with a lethal congenital malformation syndrome. Maternal-fetal medicine consultation confirmed the diagnosis and discussed the life-limiting nature of the condition. Following extensive multidisciplinary counseling, involving perinatologists and neonatologists, the couple elected to continue the pregnancy with comfort-focused palliative care planning.

At 33 weeks, she presented with preterm premature rupture of membranes and spontaneous labor. An infant was delivered vaginally, with Apgar scores of 4 at one minute and 7 at five minutes, and was immediately transferred to the neonatal intensive care unit for comfort-focused care.

**Figure 1:**
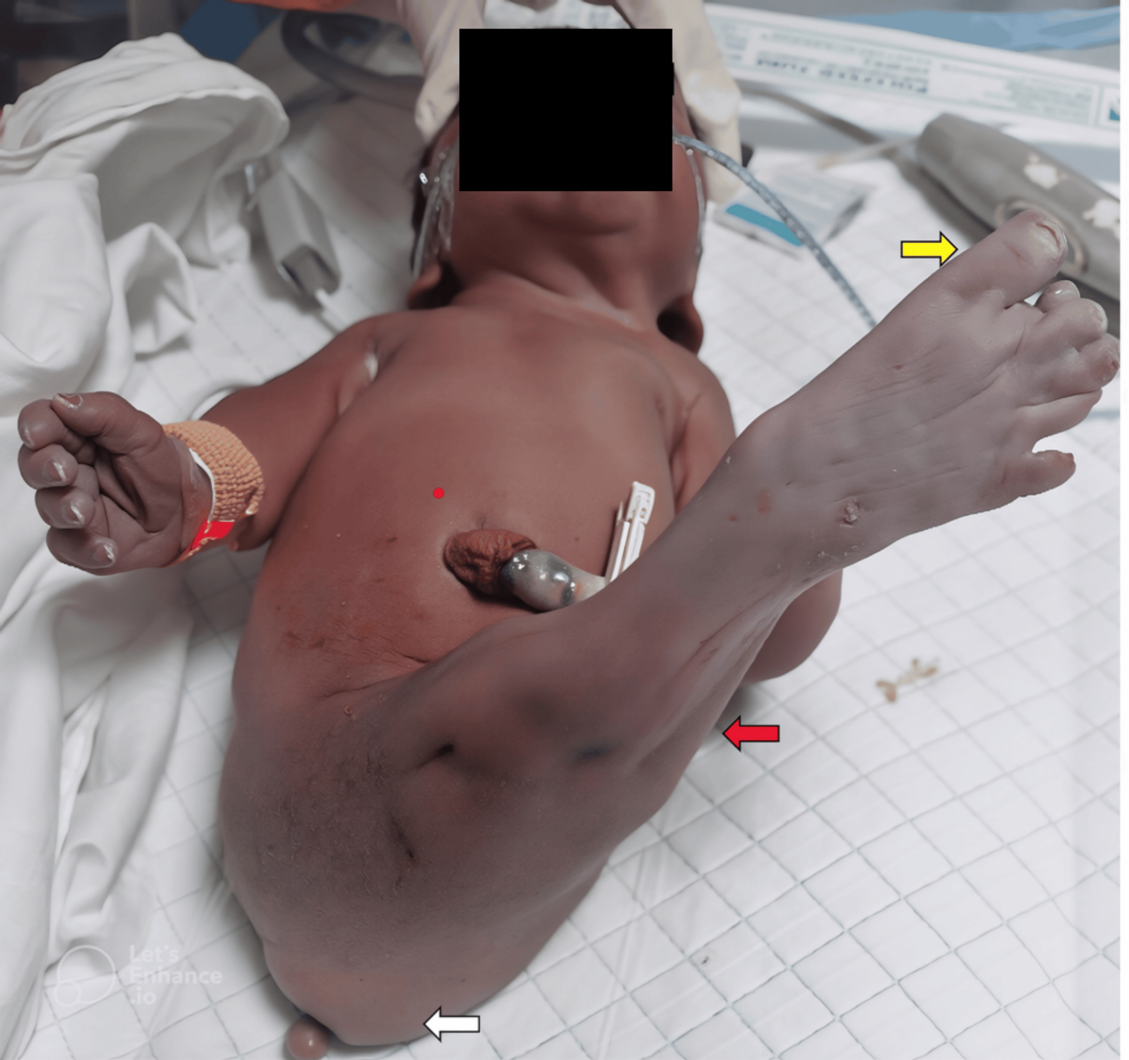
Sirenomelia features include complete lower limb fusion extending from the pelvis to the feet (red arrow), with seven toes (yellow arrow), ambiguous external genitalia, and imperforate anus (white arrow).

Physical examination revealed an appropriate-for-gestational-age preterm infant (weight 1.99 kg, length 47 cm, head circumference 29.5 cm) with multiple dysmorphic features, including low-set ears, small nose, short neck, and hyperflexed fingers. Most notably, the infant had complete fusion of the lower extremities extending from the pelvis to the feet, with seven toes total (four on the medial side and three on the lateral side), ambiguous external genitalia, imperforate anus, and a single umbilical artery (Figure 1).

Cardiovascular examination showed a heart rate of 130-142 bpm and a capillary refill time >3 seconds. Abdominal examination revealed a soft abdomen with an absent anal opening. Neurological examination showed a hypoactive state with a weak suckling reflex.

Postnatal ultrasound confirmed bilateral renal agenesis and an absent urinary bladder. Chest X-ray showed severe lung hypoplasia. Echocardiography demonstrated a structurally normal heart. Chromosomal analysis revealed a normal male karyotype (46,XY).

Given the lethal malformations and parental wishes established during prenatal counseling, comfort-focused palliative care was instituted immediately. This included gentle, non-invasive respiratory support, appropriate analgesia, maintenance of normothermia, and unrestricted family access for bonding. Despite comfort-focused palliative care, the infant died within 24 hours of life.

## Discussion

The pathogenesis of sirenomelia involves multiple complementary mechanisms operating during critical developmental windows. The defective blastogenesis theory suggests that primary failure of caudal mesoderm development during the critical gestation period (days 14-28 post-conception) results in insufficient tissue availability for normal lower body morphogenesis [6]. The vascular steal hypothesis proposes that abnormal development of the vitelline artery system results in aberrant blood flow diversion from caudal structures, leading to hypoperfusion and subsequent malformation of lower body anatomical features [7]. Contemporary molecular investigations have implicated several developmental genes in sirenomelia pathogenesis, including HLXB9, CDX1, and various members of the HOX gene family [8]. While these genetic factors establish the developmental framework for sirenomelia, environmental teratogens - particularly maternal hyperglycemia - significantly modulate pathogenic mechanisms during critical organogenesis.

While sirenomelia and caudal regression syndrome are sometimes used interchangeably in the literature, they represent distinct entities along a spectrum of caudal anomalies. Caudal regression syndrome is a broader umbrella term, encompassing various degrees of sacral agenesis and lower limb abnormalities, with highly variable severity and prognosis. Sirenomelia, by contrast, is characterized specifically by the complete fusion of the lower extremities into a single unified limb-like structure, representing the most severe and lethal end of this spectrum. The complete bilateral lower limb fusion, with seven toes and absent individual feet in our case, definitively establishes the diagnosis of sirenomelia rather than milder variants of caudal regression.

The association between maternal diabetes mellitus and sirenomelia represents a significant clinical concern. The teratogenic mechanisms likely involve hyperglycemia-induced oxidative stress, altered gene expression patterns affecting caudal mesoderm development, and resulting metabolic perturbations that compromise cellular energy metabolism [5]. These mechanisms operate synergistically during the critical period of organogenesis (gestational weeks 4-7), when caudal developmental processes and renal structures are most vulnerable to teratogenic influences.

Our case demonstrates particularly poor maternal glycemic control, with HbA1c of 9.8% at eight weeks’ gestation, 8.0% at 12 weeks, and 8.7% at 20 weeks - all well above the recommended target of <7% throughout the critical first and second trimesters [9]. These values correspond to mean plasma glucose levels exceeding 200-250 mg/dL during the crucial organogenesis window (gestational weeks 4-7), when caudal developmental processes and renal structures are most vulnerable to teratogenic influences. This persistent hyperglycemia during the periconception period and early organogenesis likely created a highly teratogenic intrauterine environment that contributed significantly to the severe phenotype observed in our patient. The coexistence of uncontrolled diabetes mellitus, sirenomelia, and a single umbilical artery further underscores the multifactorial etiologies contributing to disease severity in this case.

The phenotype observed in our case, characterized by complete fusion of the lower limbs and bilateral renal agenesis, represents the most severe end of the sirenomelia spectrum. The presence of bilateral renal agenesis is uniformly lethal, owing to associated oligohydramnios, pulmonary hypoplasia, and anuria [10]. In contrast, milder variants, with partial limb fusion and some preserved renal function, may permit limited surgical intervention and occasionally prolonged survival. However, given the irreversible nature of bilateral renal agenesis and its lethal consequences, the anatomical severity of our case predetermined a uniformly poor prognosis, regardless of potential surgical intervention for the limb anomalies.

Prenatal diagnosis remains crucial in such cases, as it facilitates early counseling and informed decision-making regarding management options. When lethal malformations, such as sirenomelia with bilateral renal agenesis, are identified, a comfort-focused care plan can be developed [10,11]. Optimal perinatal management requires a multidisciplinary approach, integrating perinatology, neonatology, genetic counseling, and psychosocial support, to guide families through these complex and emotionally challenging situations. This case exemplifies the severe phenotype of sirenomelia in the context of poorly controlled maternal diabetes mellitus and underscores critical clinical and counseling considerations for healthcare providers. It emphasizes the paramount importance of rigorous glycemic control in diabetic women during the periconceptional period and early pregnancy to prevent such devastating fetal anomalies.

## Conclusions

This case underscores the critical importance of preconceptional glycemic optimization in women with diabetes for preventing severe congenital malformations. The findings support prioritizing rigorous diabetes management before conception, and strengthening preconceptional counseling programs that address the limited therapeutic window for effective prevention. Enhanced public health initiatives, focused on diabetes education and reproductive planning for women of childbearing age with diabetes, are warranted to reduce the incidence of such outcomes.
